# Characteristics and perceived pathways of students' informal learning experiences in highly flexible public spaces of higher education: a mixed-method study based on four universities in Zhejiang Province

**DOI:** 10.3389/fpsyg.2026.1882283

**Published:** 2026-10-20

**Authors:** Juncheng Yang, Xing Yang, Yu-Pei Kuo

**Affiliations:** 1 Chinese International College, Dhurakij Pundit University, Bangkok, Thailand; 2 School of Arts and Design, Huizhou University, Huizhou, China; 3 Cross College Elite Tech Program, National Kaohsiung University of Science and Technology, Kaohsiung, Taiwan

**Keywords:** flexible public space, higher education, informal learning, learning engagement, learning satisfaction, self-efficacy, student learning experience

## Abstract

Against the backdrop of the paradigm shift toward a learning-centered model in higher education, flexible public spaces (FPS) in higher education institutions have emerged as critical campus venues supporting students' informal learning (IL). Existing studies have focused mostly on formal instructional contexts and objective spatial attributes, while operational quantitative assessment tools for Chinese university public spaces and integrated measurement of students' subjective learning experiences remain scarce. Employing an explanatory sequential mixed-methods design with a cross-sectional approach, this study examines the characteristics, internal associations, and subjective perceived pathways of students' IL in highly flexible FPS, based on 296 valid questionnaires and seven semi-structured interviews from four universities in Zhejiang Province. The study focuses on descriptive exploration and only examines statistical associations between variables; no causal inferences are drawn. Results show that students' overall IL experiences in highly flexible FPS are at a moderately high level, with significant positive correlations among learning satisfaction, self-efficacy and learning engagement. Qualitative analysis identifies three subjective perceptual pathways: atmospheric catalysis, autonomy enhancement, and social triggering, as well as an inherent tension between spatial openness and learning focus. These findings provide empirical references for the functional optimization of campus public spaces.

## 1 Introduction

In recent years, global higher education has been undergoing a sustained paradigm shift from a “teaching-centered” to a “learning-centered” approach, where learning is regarded as a contextualized and social constructed process that goes beyond the mere transmission of knowledge within classrooms. This transformation was influentially articulated by Barr and Tagg (1995). Its theoretical roots can be traced to Vygotsky's (1978) sociocultural theory, which views learning as a socially mediated, constructed, and internalized process. In recent years, amid the post-pandemic wave of restructuring in higher education, this paradigm has further intensified, driving the transformation of campus spaces from “teaching containers” to “learning ecologies” (Elkington and Dickinson, 2025). Research indicates that a significant proportion of students' learning activities take place outside the formal classroom setting, including discussions after class, collaborative work and independent exploration (Kuh, 2009a; Yau et al., 2023). Statistical evidence shows that university students spend only approximately one-fifth of their on-campus time in formal classroom learning, while most of their learning activities take place across various informal settings (Matthews et al., 2011). This kind of learning, which occurs outside the institutionalized curriculum structure and is characterized by learners' active participation, is defined as informal learning (IL) (Marsick and Watkins, 1990). It has gained increasing prominence in higher education contexts and has become a key logical starting point for the reconstruction of the learning ecosystem (Prestridge et al., 2024).

Alongside the transformation of learning modes, the functional orientation of campus spaces has also undergone profound changes. The physical environment is not a neutral container for learning; rather, it profoundly shapes learning behaviors and interaction patterns through spatial organization, resource allocation, and symbolic meaning (Oblinger, 2006; Elkington and Dickinson, 2025). Currently, campus learning spaces reviewed literature are shifting from traditional fixed classrooms to flexible, diverse, and open environments to accommodate diverse learning modes and foster student participation (Papaioannou et al., 2023). With the penetration of digital technologies, campus spaces are also exhibiting a trend toward intelligent transformation. The integration of smart technologies and physical spaces has further expanded the learning support capacity of public spaces (El Kiki et al., 2015).

In recent years, research on campus informal learning spaces has grown rapidly worldwide, giving rise to a diverse set of research directions including typological development, usage behavior analysis, and evaluation of experiential outcomes (Chin et al., 2021; Alzamil et al., 2023). However, there remain notable contextual and perspective gaps in existing literature. On one hand, most studies have focused on university contexts in Europe and North America, while research in East Asia remains dominated by case studies from select regions such as Hong Kong, with systematic empirical research in mainland China being relatively scarce (Yau et al., 2023). On the other hand, existing research has largely centered on flexible classroom design in formal instructional settings or examinations of single-type learning spaces such as libraries, and comprehensive empirical investigations of flexible public spaces (FPS) in higher education institutions remain limited. Particularly in the Chinese context, most studies remain at the conceptual level of design principles, lacking both operational quantitative assessment tools and systematic mixed-methods examinations based on students' actual learning experiences.

For the purposes of this study, campus public spaces are specifically defined as physical venues within higher education institutions that are open to all students, accessible without special access privileges, and not allocated for formal instructional purposes. These include library lobbies, student service centers, canteens, open atriums, and public corridor areas, while excluding dedicated instructional spaces such as traditional classrooms, specialized laboratories, and department-exclusive seminar rooms. When categorized by the level of support they provide for informal learning, campus public spaces fall into three tiers. The first tier consists of dedicated public learning spaces (e.g., library lobbies), which carry the strongest learning orientation and offer the highest level of learning support. The second tier comprises function-derived public spaces (e.g., student service centers, canteens). Although their primary functions are not learning-related, students spontaneously repurpose these spaces as learning settings, affording a moderate level of support. The third tier includes circulation-focused open spaces (e.g., corridors, plazas), which only support occasional, brief informal interactions and provide the lowest level of support. This study focuses on highly flexible public spaces within the first two tiers and investigates their supportive characteristics for informal learning.

Against this backdrop, grounded in a student-centered perspective, this study takes four universities in Zhejiang Province as cases, develops an evaluation framework for flexible public spaces, and explores the characteristics of students' informal learning experiences and subjective perception pathways in such highly flexible spaces. Focused on descriptive exploration rather than causal verification, the findings provide empirical support for the optimization of campus public spaces in higher education.

## 2 Literature review

### 2.1 Concept and theoretical foundations of informal learning

Informal learning (IL) is usually defined as a learning form that occurs outside the institutionalized curriculum system and lacks formal structure and evaluation mechanism. Unlike formal learning, IL does not rely on pre-set curricula or official teaching arrangements but is integrated into everyday activities and social interactions (Coombs and Ahmed, 1974). Initiated autonomously by learners, IL involves knowledge construction through cycles of action and reflection, representing a typical form of experience-based learning (Marsick and Watkins, 1990).

This classic definition laid the foundation for research in this field. Subsequent studies have gradually expanded its conceptual boundaries from feature identification and contextual empirical verification to boundary clarification. Livingstone (2006) provides a widely cited conceptualization of informal learning, emphasizing learners' autonomous control over learning content, processes, duration, and context. Studies focusing on higher education contexts by Chin et al. (2021) and Yau et al. (2023) further demonstrate that college students' informal learning is widely distributed across various campus spaces outside the classroom, encompassing diverse forms such as peer discussion, collaborative inquiry, and self-directed learning. Its value in fostering collaborative skills and problem-solving abilities may even exceed that of some formal courses. From a learning ecology perspective, Prestridge et al. (2024) systematically clarify the differences among formal learning, non-formal learning, and informal learning, and identify three core characteristics of informal learning: non-institutionalization, situational embeddedness, and self-direction.

From a theoretical standpoint, the core logic of informal learning aligns closely with the social constructivist theory of learning. Vygotsky (1978) proposed that learning is a process of knowledge construction achieved through social interaction, rather than one-way knowledge transmission. Informal learning is precisely a socially constructive process that occurs in daily social contexts and involves active participation from learners.

Although different studies have different definitions of IL, its core characteristics are basically the same: learning occurs outside the formal curriculum; Learner oriented; it is embedded in specific physical and social contexts; and learning outcomes are usually not accompanied by institutional assessment (Coombs and Ahmed, 1974; Marsick and Watkins, 1990). In this study, IL refers to students' spontaneous learning activities in the public space of colleges and universities, including group discussion type, collaborative interaction type and autonomous exploration type learning.

### 2.2 Flexible public space as a learning environment: paradigm shift and conceptual clarification

Early research on learning spaces generally regarded physical space as a neutral container for instruction. Oblinger (2006) provided a foundational systematic framework for how space profoundly shapes learning behaviors and interaction patterns through its organizational configurations, resource allocation, and symbolic meanings, laying the theoretical foundation for research on learning spaces in higher education. Alongside the paradigm shift from “teaching-centered” to “learning-centered” higher education, the functional orientation of campus spaces is undergoing systematic restructuring. Global post-pandemic research has further validated this trend, noting that learning spaces are evolving from single-function venues to diverse ecologies (Elkington and Dickinson, 2025).

Traditionally, research on campus learning spaces has largely focused on formal classrooms and library spaces. Bennett (2009) documents the shift from book-centered to learning-centered library space, and Salih et al. (2024) further demonstrate that learning commons integrate technical and service resources to support informal and social learning. In recent years, as the value of informal learning has become increasingly prominent, various public spaces such as canteens, atriums, corridors, and open plazas have gradually transcended their original functional purposes and emerged as important venues for informal learning (Chin et al., 2021; Yau et al., 2023). A state-of-the-art review by Papaioannou et al. (2023) further indicates that campus learning spaces worldwide show an overall trend of transitioning from fixed, single-function environments to flexible, multifunctional settings.

Multiple overlapping conceptualizations of learning-related spaces appear across existing scholarship, creating potential ambiguity around terminological boundaries. To clarify the analytical scope of this study, five relevant concepts are delineated hierarchically below, organized by functional purpose and physical context. This study centers exclusively on flexible public spaces (FPS) as its core unit of analysis; adjacent concepts are defined only to establish clear conceptual boundaries.

First, at the broadest functional level, informal learning spaces is an overarching functional category referring to all settings that support spontaneous, self-directed learning activities outside formal curricular structures. The concept is defined by the nature of learning activity rather than physical location, encompassing both on-campus and off-campus sites (Marsick and Watkins, 1990; Chin et al., 2021). Campus-based informal learning spaces represent the subset of these venues located within university grounds.

Second, at the physical-spatial level, campus public spaces refer to all open-access physical venues on university campuses that are not allocated for exclusive formal instructional use. Open to all students without specialized access privileges, these include canteens, atriums, lobbies, corridors, and student service centers (Chin et al., 2021). A substantial subset of campus public spaces is informally repurposed by students for learning activities, thus functioning as campus-based informal learning spaces.

Within the category of campus public spaces that support informal learning, two distinct sub-types can be distinguished by original design intent:

- Learning commons are purpose-built, learning-focused public spaces, often situated within library systems. Designed explicitly for learning support with integrated technical and service resources, they represent semi-formal, specialized public learning environments (Bennett, 2009; Salih et al., 2024).
- Flexible public spaces (FPS) are multi-purpose public venues not originally designed for exclusive educational use. They retain their primary non-learning functions (e.g., dining, student services) and are spontaneously adapted by students for informal learning, constituting a category of non-specialized informal public learning spaces (Jamieson et al., 2000; Dovey and Fisher, 2014; Chin et al., 2021).

Outside the public space framework, a further related concept is often conflated with FPS:

- Flexible learning spaces refer to reconfigurable environments within formal instructional venues (e.g., classrooms and seminar rooms), purpose-built to support structured in-class teaching activities. They fall within the formal instructional space system, defined by spatial layout convertibility to support classroom interaction (Brooks, 2011; Dovey and Fisher, 2014). As formal teaching venues, they fall outside the scope of campus public spaces.

In terms of formalization and functional specialization, the four campus-related concepts form a clear hierarchy: flexible learning spaces (formal, highest specialization) → learning commons (semi-formal, purpose-built) → flexible public spaces (informal, multi-purpose repurposed).

All three latter types may operate as informal learning spaces but differ substantially in design intent and institutional formality.

Building on this hierarchical boundary clarification, this study operationalizes FPS as its core research object: learning-supportive public environments within university public areas that exhibit spatial variability, functional versatility, and technological support capabilities, and can accommodate diverse informal learning activities. Specifically, for the purposes of this study, FPS refers to open-access public areas on university campuses with flexible layouts and multifunctional attributes, commonly used by students for informal learning activities such as self-directed study, group discussion, and collaborative exchange.

### 2.3 Five-dimensional characteristic framework of flexible public space

Research on learning spaces demonstrates that the physical and social characteristics of space shape learners' behavioral patterns and experiential perceptions (Byers et al., 2014; Salih et al., 2024). Bennett (2009) provides the historical and conceptual rationale for aligning library space with learning. In recent years, as research has gradually expanded from formal instructional spaces to IL environments, the association between spatial characteristics and learning experiences has been validated in greater detail.

Integrating core perspectives from environmental psychology, self-determination theory, and social constructivist learning theory, this study constructs a five-dimensional characteristic framework encompassing spatial variability, functional versatility, technological integration, environmental support, and social support (see Table 1). Environmental support and spatial variability focus on the shaping effects of the physical environment on learners' perceptions and behaviors; spatial variability and functional versatility can enhance learners' intrinsic motivation by satisfying their need for autonomous choice; social support and functional versatility provide the situational foundation for socially interactive learning. These five dimensions correspond to five layers of spatial attributes, physical, functional, technical, environmental, and social, respectively, realizing a stepwise implementation from macro-level theories to meso-level dimensions and micro-level indicators.

**Table 1:** Five-dimensional evaluation framework of university FPS and indicator sources.

Dimension	Observational indicator	Indicator source
Spatial variability	Furniture mobility	Dovey and Fisher (2014); Chin et al. (2021)
	Spatial separability	Chin et al. (2021);
	Layout adjustability	Dovey and Fisher (2014); Salih et al. (2024)
	Spatial boundary reconfigurability	Chin et al. (2021); Salih et al. (2024)
Functional versatility	Support for individual study	Alzamil et al. (2023); Salih et al. (2024)
	Support for group discussion	Alzamil et al. (2023); Salih et al. (2024)
	Support for leisure and relaxation	Yau et al. (2023); Wang and Han (2021)
	Support for social interaction	Salih et al. (2023); Wang and Han (2021)
Technological integration	Wireless network coverage	El Kiki et al. (2015); Salih et al. (2024)
	Adequacy of power sockets	Salih et al. (2024)
	Multimedia display equipment	El Kiki et al. (2015); Chen et al. (2022)
	Information access system	Chen et al. (2022)
Environmental support	Adequate natural lighting	Peker and Ataöv (2019); Chen et al. (2022)
	Suitable acoustic environment	Peker and Ataöv (2019); Zhang et al. (2022)
	Good thermal comfort	Zandvliet and Broekhuizen (2017); Chen et al. (2022)
	Facility comfort	Zandvliet and Broekhuizen (2017); Zhang et al. (2022)
Social support	Seating layout facilitating interaction	Salih et al. (2023); Wu et al. (2021)
	Degree of spatial openness	Salih et al. (2023); Wu et al. (2021)
	Visual connectivity	Salih et al. (2023); Wu et al. (2021)
	Social node design	Alzamil et al. (2023)

Empirical studies conducted in Chinese universities also confirm that spatial layout flexibility, environmental comfort, and functional versatility are core factors influencing students' choice of informal learning spaces (Chen et al., 2022). A systematic review by Salih et al. (2024) covering 37 empirical studies across 14 countries worldwide shows that the core design factors of informal learning spaces can be summarized into seven dimensions, including spatial design, physical settings, resource provision, and social attributes. Among these, spatial design, resources, and environmental attributes are core dimensions affecting learning experiences, which cross-validates the five-dimensional framework proposed in this study. Research on university learning spaces finds that students' perceptions of learning spaces mainly focus on comfort, flexibility, functional diversity, and support facilities (Wu et al., 2021). Scholars also note that digital technology support and social interaction opportunities are core considerations for students when selecting informal learning venues (Valtonen et al., 2021).

Spatial variability is the core physical dimension of the flexibility concept, referring to the ability to flexibly reorganize spatial layouts and enable conversion between different activity settings according to activity needs (Dovey and Fisher, 2014). Its underlying mechanism aligns with the logic of autonomy needs in self-determination theory (Ryan and Deci, 2000): adjustable spaces allow learners to independently choose their learning environments, enhancing their sense of control and intrinsic motivation. The typological study by Chin et al. (2021) confirms that movable furniture and divisible layouts can significantly improve the scene adaptability of spaces, supporting rapid switching from individual study to group discussion.

Functional versatility refers to the attribute of a single space supporting multiple activities simultaneously, associated with both autonomy satisfaction and social interaction support mechanisms. A study of pocket social spaces on campus by Alzamil et al. (2023) finds that public spaces with functional versatility can simultaneously meet diverse needs such as learning, socializing, and leisure, increasing the frequency of space use and learning stickiness. This conclusion verifies the association between the functional attributes of public spaces and learning behaviors in tropical university contexts.

Technological integration reflects the extent to which digital infrastructure supports learning activities and is an essential component of contemporary learning spaces. El Kiki et al. (2015) point out that technical elements such as wireless networks and multimedia equipment directly affect learners' duration of stay and task completion. Technological integration is no longer a standalone facility provision; rather, it is deeply integrated with spatial layout and functional design, jointly supporting flexible and diverse learning modes.

Environmental support involves physical environmental conditions such as lighting, acoustic environment, thermal comfort, and air quality, representing a concrete application of environmental perception theory in campus space contexts. The School Physical and Campus Environment Survey developed by Zandvliet and Broekhuizen (2017) takes environmental quality as a core measurement dimension. Empirical research on campus open spaces by Peker and Ataöv (2019) confirms that environmental quality significantly affects students' learning concentration and satisfaction.

Social support emphasizes the role of spatial layout in facilitating interpersonal interaction and collaboration, serving as an important spatial carrier of social constructivist learning theory. Open spatial configurations and appropriate social node design can effectively trigger peer encounters and spontaneous discussions, increasing the likelihood of informal learning (Salih et al., 2023). Li et al. (2025) corroborates this argument from the perspective of learner demand. They point out that the core value of campus third places lies in providing open and inclusive casual social scenarios, which can effectively foster students' sense of campus belonging and build a supportive social environment for informal learning activities. Public spaces with high visual connectivity, such as atriums and open halls, have a particularly pronounced social triggering effect (Wu et al., 2021). Alzamil et al. (2023) further note that the social attribute of small public spaces on campus is one of the core dimensions affecting students' social learning experiences, directly determining students' duration of stay and depth of interaction.

It should be noted that functional versatility, as the core advantage of FPS, also inherently limits support for deep cognitive processing. With diverse original functions, public spaces cannot be designed with the same level of strict acoustic isolation and spatial partitioning as dedicated study rooms. Accordingly, they are better suited to low-focus, surface-level learning scenarios such as routine independent study and social interaction and have inherent limitations in supporting higher-order cognitive tasks that require deep concentration and complex deliberation. This characteristic essentially reflects students' general dual demand for informal spaces: students require both open social interaction settings and private focused study spaces, and the two cannot be simultaneously maximized within a single undifferentiated space.

### 2.4 Three-dimensional measurement constructs of informal learning experiences

When examining IL experiences in flexible public spaces, existing studies typically select core variables for measurement across three dimensions: affective, cognitive, and behavioral (Fredricks et al., 2004).

Specifically, the affective dimension corresponds to learning satisfaction (emotional evaluation of the learning experience), the cognitive dimension corresponds to self-efficacy (beliefs about one's own learning competence), and the behavioral dimension corresponds to learning engagement (actual investment of time and effort in learning activities). Among these, learning satisfaction, self-efficacy, and learning engagement are the three most widely adopted indicators.

Learning satisfaction refers to a multifaceted positive affective response to the learning process and outcomes, reflecting the perceived fulfillment of learning desires and the match between learning experience and personal expectations (Topala and Tomozii, 2014; She et al., 2021). Existing research indicates that learning satisfaction is closely associated with stronger learning motivation and positive learning-related outcomes, serving as a core affective indicator for measuring the quality of learning experiences (Wu et al., 2015; Gopal et al., 2021).

Self-efficacy, which derives from social cognitive theory, refers to an individual's confidence in their ability to perform specific learning tasks; it manifests as a sense of self-assurance regarding the acquisition of knowledge, problem-solving and the achievement of learning objectives (Bandura, 1997). Higher self-efficacy can enhance engagement in learning, foster a positive attitude toward learning, and increase the likelihood of achieving learning objectives (Rorimpandey and Midun, 2021; Shea and Bidjerano, 2010). In the context of spatial studies, supportive physical and social environments can indirectly improve learning outcomes by enhancing learners' perceptions of their own competence.

Learning engagement is a multidimensional construct encompassing behavioral, emotional and cognitive dimensions (Fredricks et al., 2004). Behavioral engagement manifests as the actual investment of time and effort in learning activities; affective engagement reflects interest in and a sense of belonging toward learning activities; cognitive engagement involves deep cognitive processing and the application of learning strategies (Fredricks et al., 2004). In higher education research, learning engagement is often defined as the overall level of time and effort students devote to academic activities (Kuh, 2009a). Astin (1999) points out that the intensity of student engagement is a key factor influencing learning outcomes. Empirical research indicates that high levels of engagement are significantly associated with academic achievement, knowledge retention and graduation rates (Carini et al., 2006).

Overall, learning satisfaction, self-efficacy, and learning engagement represent the core variables at the three levels of emotional evaluation, confidence in one's abilities and behavioral engagement, respectively. There is a significant correlation among these three factors, and they collectively influence learning outcomes and the willingness to continue learning (Badiozaman et al., 2023). However, existing research validating the associations among the three has largely focused on formal curricula and online learning contexts. In the context of informal learning in flexible public spaces of higher education, the characteristics and interrelationships of the three dimensions still lack systematic empirical examination.

### 2.5 Limitations of existing research

Although the association between learning spaces and students' informal learning experiences has received extensive scholarly attention, four core limitations remain in existing research when viewed from the perspective of the present study:

At the assessment tool level: there is a lack of quantitative evaluation frameworks for flexible public spaces in higher education institutions. Existing research has made notable progress in typological development: Chin et al. (2021) proposed a four-category classification of informal learning spaces based on Hong Kong universities, and Salih et al. (2024) further constructed a global typological framework of informal learning spaces encompassing six categories through a systematic review covering 14 countries, clarifying the boundaries of learning support characteristics across public spaces of different functional origins. However, the body of research overall remains at the level of qualitative classification. No systematic, operable quantitative tool for the flexibility characteristics of public spaces has yet been established, making it difficult to support site selection, cross-case comparison, and large-scale empirical testing. Meanwhile, existing studies mostly explore the physical attributes of campus open spaces from a spatial design perspective (Peker and Ataöv, 2019) or focus on the functional development of intelligent interactive environments (El Kiki et al., 2015), and few derive optimization pathways from the bottom up through the lens of learners' subjective experiences.

At the research context level: relevant studies in mainland China have grown gradually in recent years, yet they still exhibit clear limitations in perspective and methodology. Most existing international studies focus on European and North American university contexts, while East Asian research remains dominated by case studies from select regions such as Hong Kong, China (Chin et al., 2021; Yau et al., 2023). Southeast Asian scholarship has also built a body of typological and influencing-factor research on campus public spaces (Alzamil et al., 2023). Domestic studies in China have preliminarily verified the influence of spatial design characteristics on students' learning space choices (Chen et al., 2022; Wang and Han, 2021), but most adopt a spatial design perspective and emphasize objective descriptions of physical attributes. Cross-contextual visual preference survey studies further confirm that the demand for spatial attributes of informal learning spaces has cross-cultural consistency (Salih et al., 2023). Systematic mixed-methods investigations with multi-dimensional integrated measurement of students' informal learning experiences in public spaces of mainland Chinese universities remain relatively scarce, and the generalizability of findings across different cultural and campus governance contexts requires further verification.

At the experience integration level: systematic research on multi-dimensional learning experiences is insufficient. Existing studies largely center on formal instructional scenarios or single experiential variables (Brooks, 2011). As Fredricks et al. (2004) argue, learning experience is multidimensional, encompassing behavioral, emotional, and cognitive aspects; however, these dimensions are often examined in isolation rather than in an integrated manner, and informal or flexible public spaces remain underexplored. Therefore, a more integrated approach that simultaneously examines satisfaction, self-efficacy, and engagement in diverse spatial contexts is urgently needed.

At the practical optimization level: there is a lack of bottom-up optimization pathways informed by the student perspective. Most existing design recommendations for informal learning spaces are derived from architectural case studies and professional design judgment (Salih et al., 2024), and few derive actionable optimization directions from learners' real-world experience feedback. Existing research on smart campus spaces mostly focuses on technical facility configuration and spatial design optimization (El Kiki et al., 2015), and likewise rarely extract optimization pathways from the bottom up through learners' subjective experiences. As Oblinger (2006) observed, space itself does not create learning; what truly matters is how learners perceive, use, and assign meaning to space. Accordingly, exploring spatial experiences and optimization pathways from a student-centered perspective carries important practical value.

Building on these gaps, this study advances existing research in three specific dimensions:

- Instrument development: it develops a 5-dimensional, 20-item quantitative evaluation checklist for university FPS with preliminary reliability testing, addressing the lack of operational assessment tools for public space flexibility in mainland China.
- Empirical complement: it adopts a mixed-methods design to systematically describe the three-dimensional informal learning experiences (satisfaction, self-efficacy, engagement) of students in highly flexible FPS in mainland Chinese universities, supplementing the empirical evidence base for East Asian contexts.
- Perspective expansion: it induces three subjective experiential pathways from the student perspective, providing bottom-up perceptual evidence for spatial optimization, which complements the dominant top-down design perspective in existing research.

### 2.6 Research question

Building on the research gaps and centered on the core theme of flexible public spaces and students' informal learning experiences in higher education, this study formulates one main research question and four supporting research questions.

#### 2.6.1 Main research question

What are the overall characteristics of students' informal learning experiences in highly flexible public spaces in higher education, and what subjective experiential pathways can be induced from interview data to offer perceptual interpretations of these characteristics?

#### 2.6.2 Supporting research questions

RQ 1 (Foundational level): what are the core structural characteristics of the selected highly flexible university FPS, and what is the inter-rater reliability performance of the evaluation checklist developed in this study?

RQ 2 (Core descriptive level): what is the overall level of students' informal learning experiences in the selected typical FPS?

RQ 3 (Associative mechanism level): is there a significant statistical correlation among learning satisfaction, self-efficacy, and learning engagement?

RQ 4 (Practical extension level): what are the feasible optimization directions for university FPS based on students' experiential feedback?

## 3 Materials and methods

### 3.1 Research design and mixed-methods strategy

This study employs an explanatory sequential mixed-methods design (Saunders et al., 2016), implemented in two sequential phases. Phase 1 (Spatial assessment and case site screening): the research team conducted on-site field observations of campus public spaces to evaluate their flexibility characteristics and identify representative typical FPS for formal data collection. Phase 2 (Quantitative survey followed by qualitative interpretation): a cross-sectional questionnaire survey was first administered to capture the overall quantitative profiles of students' informal learning experiences. A semi-structured interview protocol was then developed based on preliminary questionnaire results, and qualitative data were collected to interpret and complement the quantitative findings, which achieves methodological triangulation across data sources.

Design rationale: as an exploratory investigation, this study prioritizes highly flexible sites as representative cases to focus on characterizing experiential features and perceived pathways among students in these spaces. Subsequent studies may include medium- and low-flexibility control groups to enable comparative analysis. The full research workflow is summarized in Figure 1.

**Figure 1:**
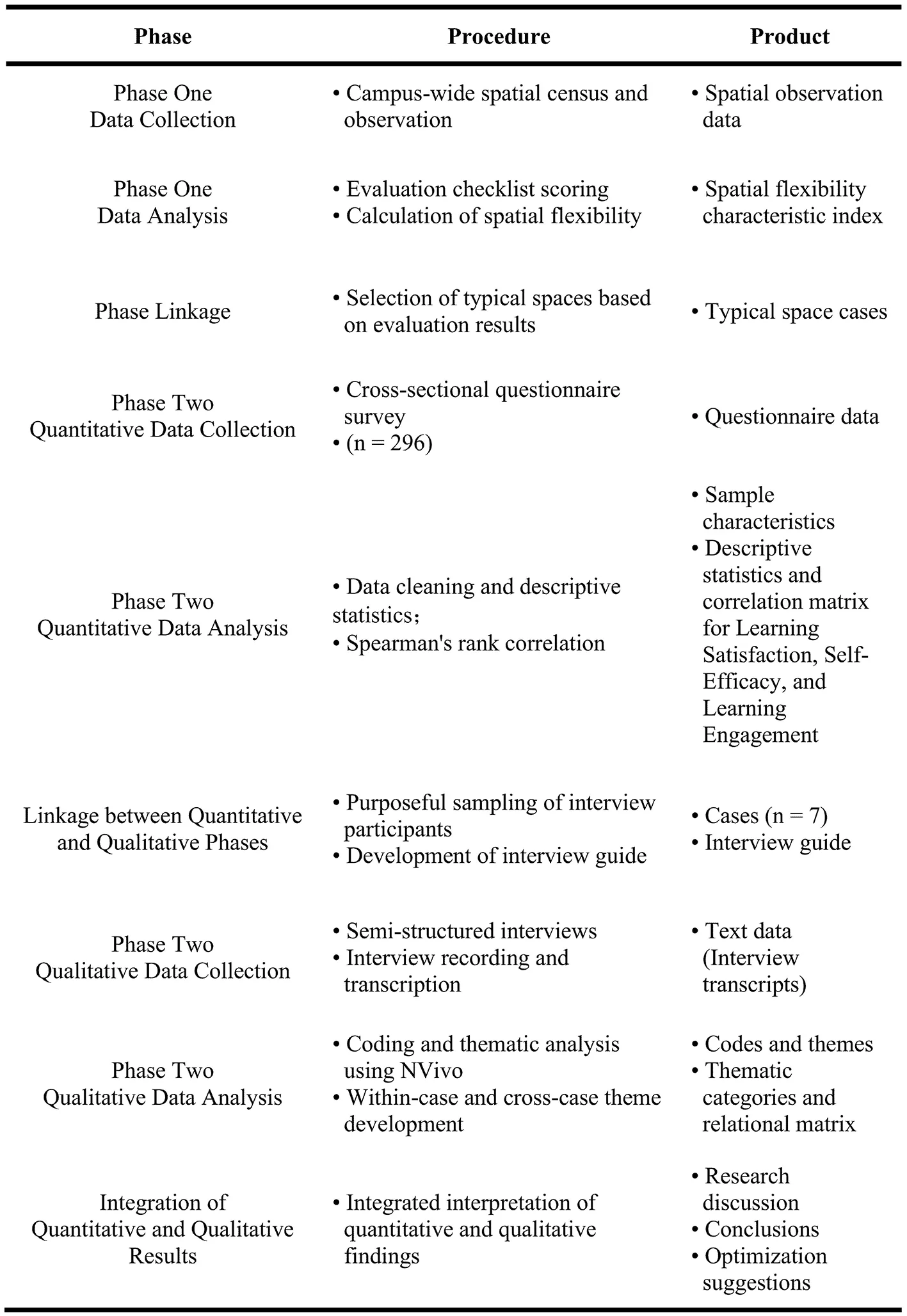
Research design process for mixed-methods studies. The study was conducted in two phases: phase 1 involved spatial assessment and screening of flexible public spaces; Phase 2 included questionnaire surveys and semi-structured interviews to examine students' informal learning experiences.

### 3.2 Research setting and case selection

Between September and October 2025, the research team conducted field investigations at four universities of different types in Zhejiang Province. ZN is a comprehensive university with a student and staff population of approximately 30,000 and a campus area of about 220 hectares; ZT is a science and engineering university with approximately 6,000 students and staff and a campus area of 32 hectares; HU is a normal university with around 18,000 students and staff and a campus area of 104 hectares; CA is an arts university with roughly 7,000 students and staff and a campus area of 53 hectares. Each institution has distinct educational orientations and campus spatial configurations, providing representative contexts for the study. All four universities offer students access to various types of public spaces, including library lobbies, student service centers, and canteens, providing foundational conditions for conducting FPS research.

In line with the three-tier classification of campus public spaces introduced in the Introduction, this study focuses on highly flexible public spaces in the first two tiers (dedicated public learning spaces and function-derived public spaces). Accordingly, the inclusion and exclusion criteria for study sites are defined as follows.

Inclusion criteria for study sites: first, non-classroom public venues open to all students; second, spaces where students can spontaneously engage in informal learning; third, spaces that can be fully observed using the evaluation checklist.

Exclusion criteria for study sites: department-exclusive spaces and closed reservation-based seminar rooms (lacking full public accessibility); outdoor open plazas (strongly influenced by weather and seasonal conditions, unable to stably support sustained informal learning); and circulation-focused open spaces in the third tier, which only support occasional brief interactions.

Case selection process: this study focuses on students' experiences in highly flexible public spaces. Accordingly, a flexibility score of ≥80% was pre-defined as the inclusion threshold for highly flexible FPS at the study design stage. During the field investigation, census sampling was first applied to 12 major public sites across the four universities (three sites per university) that met the inclusion and exclusion criteria. Each site was scored on-site using the evaluation checklist. The sites were then ranked in descending order of flexibility score, and the top three sites that met the threshold were selected as study cases: the Sunlight Hall Student Service Center at ZT University (90% flexibility), Herun Canteen at HU University (85% flexibility), and the Library Lobby at ZN University (80% flexibility).

All evaluated sites at CA Arts University fell below the 80% threshold. To ensure baseline homogeneity across cases and avoid confounding the analysis of experiential characteristics, CA University sites were not included in the formal study sample and only served as part of the initial site screening census.

This cross-institutional, multi-type case design was adopted to cover FPS configurations across different educational contexts and enhance the generalizability of findings. Selecting three sites within a single university would expose the sample to confounding effects from uniform campus management practices, student population characteristics, and campus cultural atmosphere, making it difficult to isolate the effects of spatial attributes themselves. Selecting different types of sites across multiple universities can, to a certain extent, control for homogeneity bias at the institutional level and improve the external validity of conclusions.

The three case sites represent three typical types of campus public spaces: student service center, university canteen, and library lobby. They correspond to three widely observed function-originated categories of campus informal learning spaces: library-public spaces, catering-derived spaces, and service-derived spaces (Cunningham and Walton, 2016). They also cover the two core categories of public informal learning spaces in Salih et al.'s (2024) typology: public indoor and semi-public indoor spaces. Specifically, the ZN University Library Lobby is a semi-public indoor space tied to the dedicated learning function of the library, with clear functional boundaries. The ZT Sunlight Hall and HU Herun Canteen are public indoor spaces with no exclusive functional access restrictions, supporting a diverse range of activities. The type of representativeness is sufficient, and their spatial characteristics correspond to the five dimensions of FPS as follows:

ZT Sunlight Hall: located on the first floor of the Student Service Center in the central campus area, this open-plan hall measures approximately 32 m in length and 16 m in width, totaling around 510 m^2^ with no fixed wall partitions. It is open from 8:00 to 22:00 on weekdays, serving all students for daily affairs, leisure communication and spontaneous learning. A large skylight in the ceiling provides abundant natural light and creates a highly open spatial atmosphere. All interior furniture is movable and reconfigurable, allowing free arrangement into three modes: individual study zones, group discussion zones, and collective activity areas. Wooden bookshelves run longitudinally along the inner perimeter of the central atrium, situated within the main hall and acting as flexible spatial dividers. The space is equipped with Wi-Fi, sufficient power outlets, and partial multimedia screens. The seating layout supports both solitary study and group interaction, with prominent spatial variability and social support (see Figure 2).

**Figure 2:**
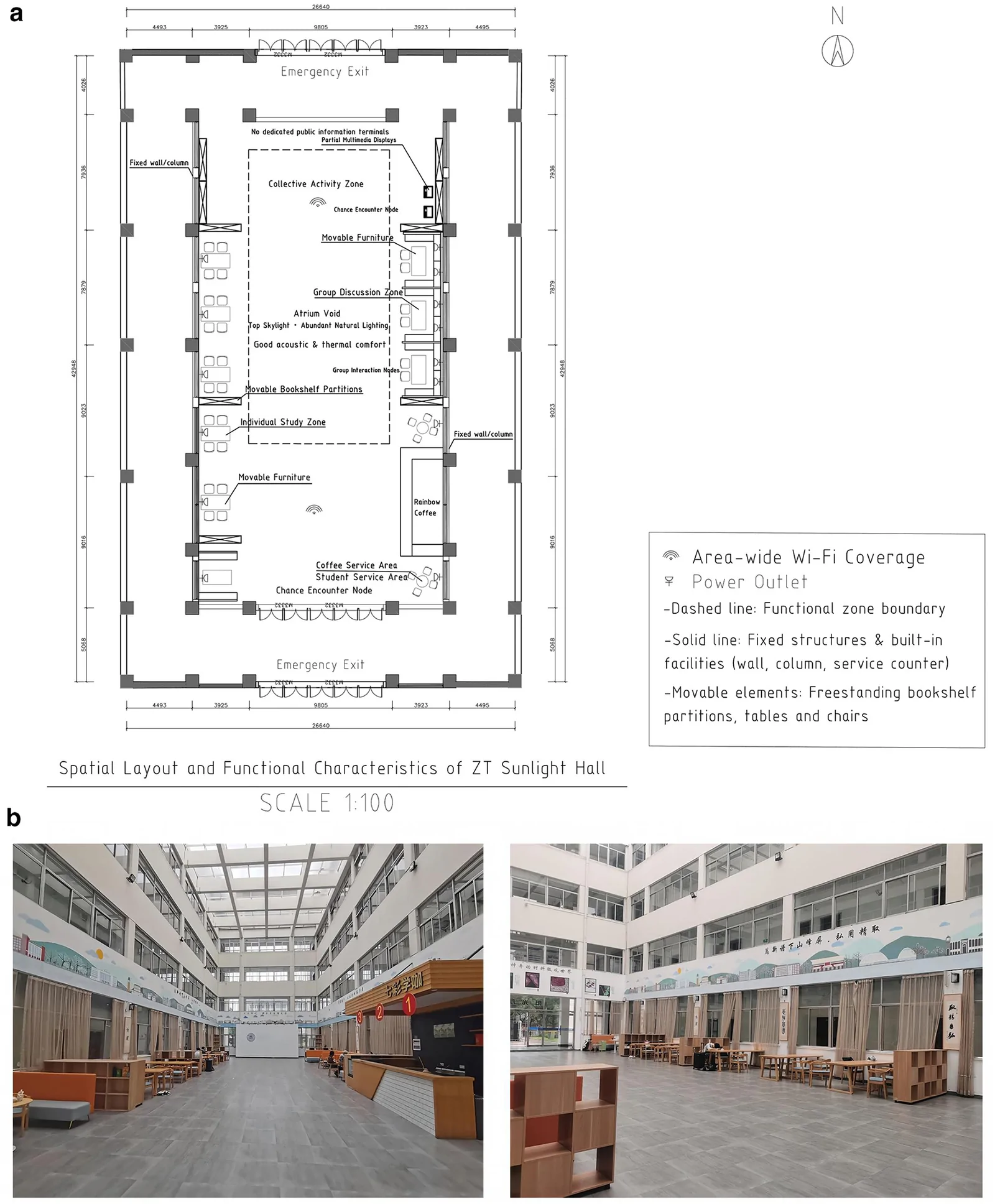
Spatial layout and site views of ZT Sunlight Hall. **(a)** Schematic floor plan illustrating the spatial structure, functional zoning, and spatial attribute configuration; **(b)** On-site photographs showing the actual interior environment and usage scenarios. This open-plan hall in the student service center adopts a barrier-free rectangular layout with fully movable furniture and flexible perimeter divisions, demonstrating strong spatial variability and social support.

HU Herun Canteen: located on the first floor of the canteen on the East Campus, this public dining area covers approximately 1,500 m^2^. It is open from 6:30 to 21:00 daily, primarily serving dining needs while also supporting spontaneous learning activities during non-peak hours. Three functional zones naturally emerge by usage pattern: a long-table zone, a booth seating zone, and a curved sofa zone. There are no fixed wall partitions between zones, representing functional zoning rather than physical spatial separation. The booth seating zone features fixed integrated booths that cannot be moved; tables and chairs in the long-table zone and curved sofa zone can be freely rearranged to form various configurations such as single-person corner seats and multi-person round tables. The space is equipped with basic Wi-Fi and power outlets but lacks large multimedia equipment. Foot traffic is high during meal hours, and natural lighting is moderate, but the site exhibits prominent functional versatility (see Figure 3).

**Figure 3:**
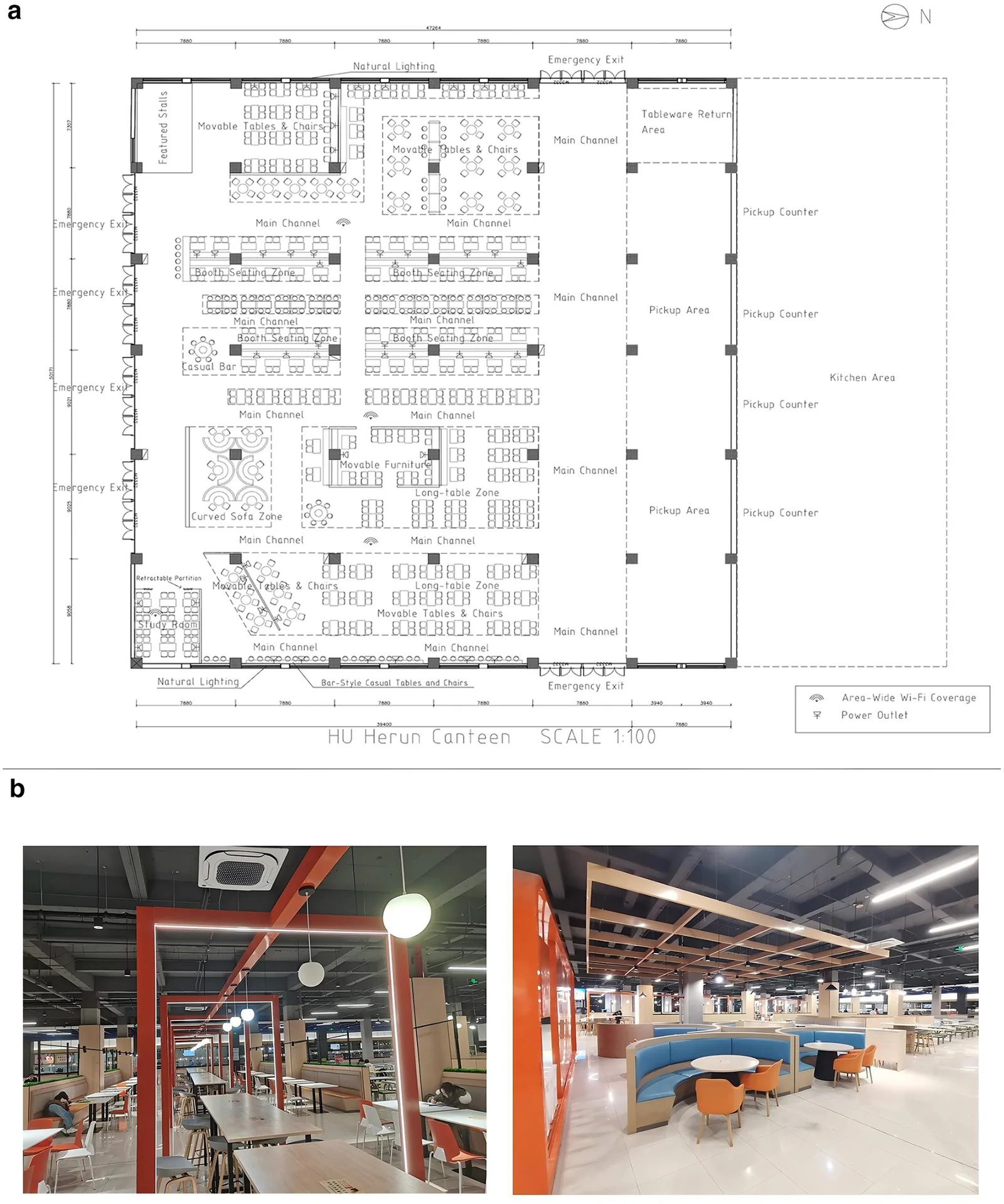
Spatial layout and site views of HU Herun Canteen. **(a)** Schematic floor plan illustrating the spatial structure, functional zoning, and fixed/movable element distribution; **(b)** On-site photographs showing the actual dining-learning composite space and seating forms. This public dining area of the university canteen forms three functional zones without fixed wall partitions, with partially reconfigurable seating and prominent functional versatility.

ZN Library Lobby: located at the main entrance atrium of the campus central library, this space covers approximately 960 m^2^, with movable furniture in the window-side rest area. It is open consistent with library hours (7:30–22:30 daily), serving as a transitional and rest space for library users. The space is fully equipped with internet access, power outlets, and multimedia information terminals. The acoustic, lighting, and thermal environmental conditions are of high quality. There are few dedicated social nodes, but the overall open layout and high visual permeability give space prominent technological integration and environmental support (see Figure 4).

**Figure 4:**
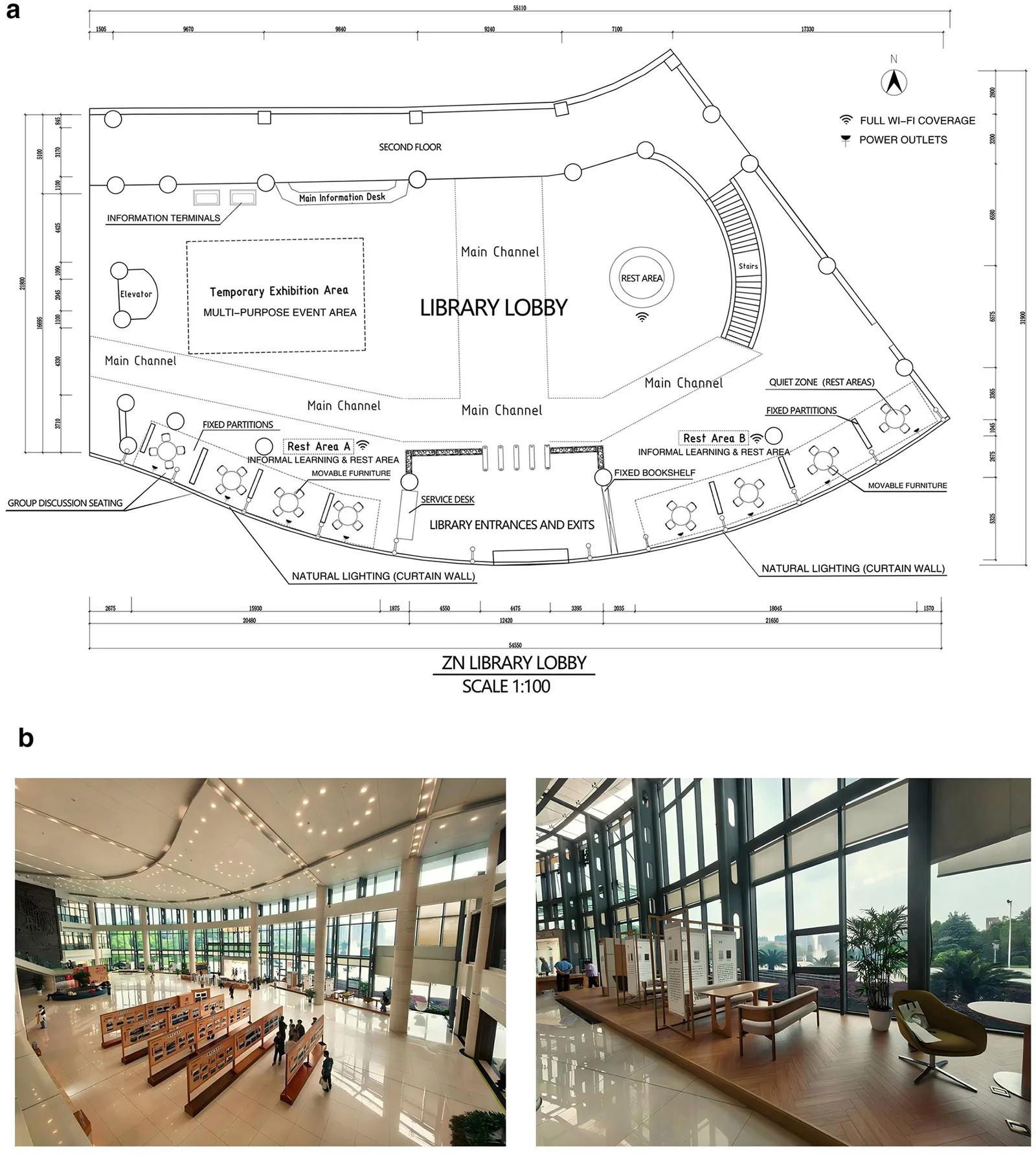
Spatial layout and site views of ZN Library Lobby. **(a)** Schematic floor plan illustrating the spatial structure, functional zoning, and facility configuration; **(b)** On-site photographs showing the actual atrium environment and rest areas. This atrium at the library entrance is equipped with comprehensive technical infrastructure and high-quality acoustic, lighting and thermal environments, exhibiting strong technological integration and environmental support.

### 3.3 Research instruments

#### 3.3.1 Flexible public spaces evaluation checklist

Based on the five-dimensional framework outlined above, the *University FPS Characteristic Evaluation Checklist* was developed, comprising a total of 20 binary observational indicators (see Table 2). The instrument development and quality control procedures are described as follows.

**Table 2:** Checklist for evaluating FPS features in higher education institutions.

Dimension	Observational indicator	Spatial coding (Yes = 1, No = 0)
Spatial variability	Furniture mobility	□ Yes □ No
	Spatial separability	□ Yes □ No
	Layout adjustability	□ Yes □ No
	Spatial boundary reconfigurability	□ Yes □ No
	Subtotal	____/4
Functional versatility	Support for individual study	□ Yes □ No
	Support for group discussion	□ Yes □ No
	Support for leisure and relaxation	□ Yes □ No
	Support for social interaction	□ Yes □ No
	Subtotal	____/4
Technological integration	Wireless network coverage	□ Yes □ No
	Adequacy of power sockets	□ Yes □ No
	Multimedia display equipment	□ Yes □ No
	Information access system	□ Yes □ No
	Subtotal	____/4
Environmental support	Adequate natural lighting	□ Yes □ No
	Suitable acoustic environment	□ Yes □ No
	Good thermal comfort	□ Yes □ No
	Facility comfort	□ Yes □ No
	Subtotal	____/4
Social support	Seating layout facilitating interaction	□ Yes □ No
	Degree of spatial openness	□ Yes □ No
	Visual connectivity	□ Yes □ No
	Social node design	□ Yes □ No
	Subtotal	____/4
	Total score (/20)	____/20
	Flexibility level (%)	____ %

1 = has this characteristic; 0 = does not have this characteristic.

Indicator screening process: first, commonly used spatial characteristic indicators from existing studies (Dovey and Fisher, 2014; Chin et al., 2021; Peker and Ataöv, 2019) were systematically reviewed and categorized into the five dimensions to form a preliminary draft. Subsequently, three experts in the fields of higher education research and educational architecture design were invited to independently review the accuracy of indicator wording and the rationality of dimensional assignment. Ambiguous indicators were removed, and the dimensional classification logic was adjusted to produce a revised version of the evaluation checklist.

Pilot evaluation: prior to formal field data collection, the research team selected five different types of public spaces at one university to conduct a pilot evaluation. The purpose was to standardize the judgment criteria and observational definitions for each indicator, ensuring consistency in scoring standards. Following the pilot evaluation, 20 formal evaluation indicators were finalized.

Quality control: each case site was independently scored on-site by two researchers who had received standardized training. In the event of scoring discrepancies, the two researchers returned to the site for joint re-observation and reached a consensus through thorough discussion.

Scoring rules:

- Single indicator scoring: each of the 20 indicators is scored dichotomously: 1 if the characteristic is present, 0 if absent.
- Dimensional score: sum of scores for the four indicators within each dimension (full score = 4 per dimension).
- Total score: sum of scores across all five dimensions (full score = 20).
- Flexibility percentage: calculated as (actual total score / maximum possible score of 20) × 100%. A higher percentage indicates stronger flexible public space attributes of the venue.

Since no empirically validated differentiated weighting scheme for indicators has been established in the field to date, this exploratory study assigns equal weight to all 20 indicators.

#### 3.3.2 Student questionnaire

The core latent variables of the questionnaire were adapted from three internationally validated scales: learning Satisfaction (Wang, 2003), Self-Efficacy (Zimmerman and Kulikowich, 2016), and Learning Engagement (Kuh, 2009b). The original scales were developed in online learning contexts.

The conceptual rationale for selecting these three specific scales is grounded in the classic three-dimensional framework of learning experience proposed by Fredricks et al. (2004), which conceptualizes learning experience as a holistic construct comprising affective, cognitive, and behavioral dimensions. This framework is a well-validated paradigm in educational psychology for measuring situated learning experiences and is highly applicable to the assessment of informal learning experiences in public space contexts.

- The affective dimension corresponds to the Learning Satisfaction scale (Wang, 2003). This scale focuses on learners' overall emotional evaluation of the learning environment and is a mature instrument for measuring situational learning experience satisfaction, which precisely matches the demand for evaluating environmental experience in FPS contexts.
- The cognitive dimension corresponds to the Self-Efficacy scale (Zimmerman and Kulikowich, 2016). Developed based on social cognitive theory, this scale measures learners' beliefs about their own learning competence, corresponding to the dimension of perceived self-regulated learning ability in FPS settings.
- The behavioral dimension corresponds to the Learning Engagement scale (Kuh, 2009b). This scale focuses on learners' behavioral investment and is a widely used standard instrument for measuring learning participation in higher education, which is suitable for measuring learning behavioral engagement in FPS contexts.

This three-dimensional theoretical framework directly drove the item development process for the flexible public space context. The specific item distribution is presented below.

For this study, the adaptation followed three core principles and procedures:

- Construct retention: the theoretical definitions and core measurement kernels of the three constructs were fully retained; only the situational carrier of the items was adjusted.
- Contextual adaptation: all situational references to “online learning platform/course” in the original scales were uniformly replaced with “this flexible public space”, while the semantic direction and scoring logic of the items remained unchanged.
- Item screening: Two experts in higher education research independently reviewed the contextual appropriateness of each item. Two items irrelevant to public space scenarios (e.g., items related to course assignment submission in the original scales) were removed.

A preliminary phrasing pre-test with three university students was then conducted to verify the clarity, readability and contextual appropriateness of item wording. This small-sample pretest only focused on language expression optimization and did not involve psychometric testing of the scale.

The final instrument includes 17 items: six for learning satisfaction, five for self-efficacy, and six for learning engagement.

Following completion of the initial questionnaire draft, content and wording were first revised for contextual fit. Two experts in higher education research independently reviewed the contextual appropriateness and wording accuracy of each item. Items inconsistent with campus public space scenarios were removed, and ambiguous phrasing was revised. Three currently enrolled university students then completed a pilot test. Based on their feedback, the naturalness and clarity of wording were further refined, resulting in the final survey instrument. The formal questionnaire includes five modules: demographic information, learning satisfaction, self-efficacy, learning engagement, and interview invitation. A 5-point Likert scoring format was used, with 1 = strongly disagree and 5 = strongly agree.

Cronbach's alpha coefficient was used to test the internal consistency reliability of each dimension. It should be noted that Cronbach's alpha only reflects internal consistency among items and does not indicate the construct validity of the scale. Confirmatory factor analysis (CFA) was not conducted in this study, as the questionnaire represents an exploratory measurement instrument adapted for the local context.

#### 3.3.3 Semi-structured interviews

Consistent with the logic of the explanatory sequential mixed-methods design, the interview guide was developed based on high- and low-scoring items from the preliminary questionnaire survey. It was organized around four core themes: characteristics of learning activities, space use preferences, experiential perceptions, and optimization suggestions. Qualitative data were used to complement and elaborate on the quantitative results.

The four thematic modules were designed to align with the three core constructs of the questionnaire:

- Characteristics of learning activities and experiential perceptions directly correspond to learning satisfaction, self-efficacy, and learning engagement, and are used to interpret and triangulate the quantitative findings.
- Space use preferences complement the analysis of the relationship between spatial attributes and learning experiences.
- Optimization suggestions provide a practical basis for FPS optimization.

Interviewees were selected via purposive sampling from students who volunteered at the end of the questionnaire, with variation across grade level, academic discipline, and space use patterns. A total of 7 interviews were completed, with thematic saturation serving as the sampling stopping criterion. Interviews were conducted in Chinese, lasted 20–30 minutes each, were fully audio-recorded, and transcribed verbatim. Original audio recordings were deleted after analysis, and all respondent information was fully anonymized.

Coding was conducted independently by two researchers trained in qualitative research methods. The coding process followed the standard sequence of open coding, axial coding, and thematic refinement. Disagreements in coding were resolved through thorough discussion and consensus between the two coders. As an exploratory analysis centered on interpretive meaning, this study employed consensus-based reliability testing rather than Kappa coefficients, a standard practice in qualitative educational research (Strauss and Corbin, 1990). Coding and data management were performed using NVivo 11 software.

### 3.4 Data analysis methods

This study adopts an exploratory explanatory sequential mixed-methods design. Data analysis progresses sequentially along the logic of characteristic description – correlation testing – qualitative complementation, aligned with the two-phase research objectives. Specific methods are as follows:

#### 3.4.1 Spatial assessment analysis

Spatial assessment data were used to calculate the flexibility characteristic index for each space based on indicator scores, which were then converted into percentages to compare the levels of flexible characteristics across different FPS.

#### 3.4.2 Questionnaire statistical analysis

Data analysis was performed using SPSS 25.0 software. Two main statistical methods were employed: descriptive statistics and Spearman's rank correlation analysis. Descriptive statistics were used to present means, standard deviations, and frequency distributions of each variable, to reveal basic data trends and group characteristics. Spearman's rank correlation analysis was used to test the associations among learning satisfaction, self-efficacy, and learning engagement, to address RQ3.

As an exploratory study, the core goal is to identify the experiential characteristics and subjective perception pathways of highly flexible public spaces. Descriptive statistics and rank correlation analysis are sufficient to address the preset research questions. Future research may adopt a longitudinal tracking design and add high- and low-flexibility control groups to further examine the causal relationships and mechanisms between spatial characteristics and learning experiences.

#### 3.4.3 Qualitative analysis of semi-structured interviews

This study employed thematic analysis to systematically code interview transcripts. The analysis of interview data included four steps: transcription, coding, analysis, and interpretation. Systematic coding and categorization were conducted using NVivo 11 software, and coding categories were rigorously revised to ensure no redundancy (Strauss and Corbin, 1990). The coding process involved multiple rounds of iteration, and all units of meaning retained the psychological integrity of the expressed views; they were neither fragmented into meaningless segments nor conflated across different thematic perspectives (Ratner, 2002, p. 169). Ultimately, core study findings were distilled through thematic analysis, which served to complement, interpret, and triangulate the quantitative results.

## 4 Research results

This study adopts an explanatory sequential mixed-methods design. Three typical flexible public spaces, all with flexibility scores between 80% and 90%, were screened through on-site spatial assessment. The second phase integrates questionnaire surveys and semi-structured interviews to systematically investigate students' informal learning experiences. The core findings and logical framework of the two-phase study are depicted in Figure 5.

**Figure 5:**
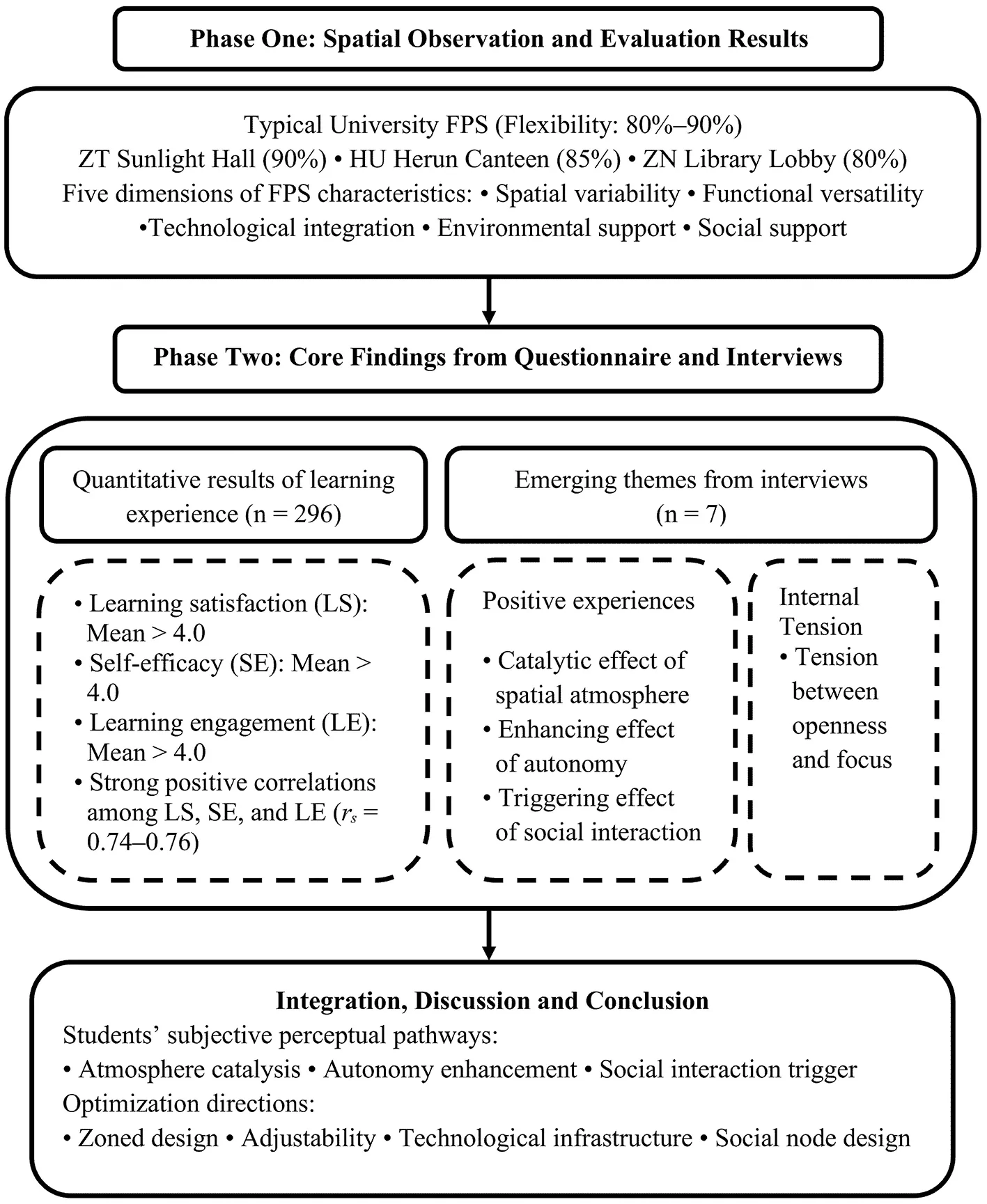
Two-phase mixed-methods framework and core research findings. The framework presents the flexibility levels of three typical flexible public spaces from Phase 1, and summarizes the descriptive characteristics of learning experiences, inter-dimensional correlation patterns, subjective experiential pathways, and the core openness-concentration tension obtained from Phase 2.

### 4.1 Spatial assessment results and instrument reliability

In the first phase of the study, three typical FPS sites were selected from four universities following spatial assessment: ZT Sunlight Hall (90%), HU Herun Canteen (85%), and ZN Library Lobby (80%). The assessment was conducted strictly in accordance with the 20 theoretical indicators across five dimensions specified in Table 1. On-site item-by-item scoring was performed using the field evaluation checklist in Table 2, and the dimensional scores and overall flexibility scores were aggregated as presented in Table 3.

**Table 3:** Summary table of typical university FPS assessments.

Dimension	HU herun canteen	ZT sunlight hall	ZN library lobby
Spatial variability	4	3	2
Functional versatility	4	4	3
Technological integration	2	3	4
Environmental support	3	4	4
Social support	4	4	3
Total score (/20)	17	18	16
Flexibility level (%)	85%	90%	80%

Flexibility level = (total score/20) × 100%. A higher percentage indicates stronger FPS characteristics.

#### 4.1.1 ZT sunlight hall

- Spatial variability: 3/4. Features movable furniture and partial flexible partitions, but spatial boundary reconfigurability is limited.
- Functional versatility: 4/4. Fully supports individual study, group discussion, leisure relaxation, and social interaction.
- Technological integration: 3/4. Equipped with Wi-Fi and sufficient power outlets, with partial multimedia display equipment.
- Environmental support: 4/4. Excellent natural lighting from the skylight, good acoustic control, and comfortable thermal environment.
- Social support: 4/4. Open layout with high visual connectivity, and seating layouts that facilitate interaction.

#### 4.1.2 HU Herun canteen

- Spatial variability: 4/4. Full furniture mobility and layout reconfigurability in the long-table and curved sofa zones.
- Functional versatility: 4/4. Covers learning, dining, leisure, and social interaction scenarios.
- Technological integration: 2/4. Only basic Wi-Fi and power outlets; lacks multimedia and information access systems.
- Environmental support: 3/4. Moderate natural lighting; acoustic environment is affected by meal-time foot traffic.
- Social support: 4/4. Diverse seating forms support both independent use and group interaction.

#### 4.1.3 ZN library lobby

- Spatial variability: 2/4. Movable furniture in the rest area, but overall layout is relatively fixed.
- Functional versatility: 3/4. Primarily supports study and discussion; limited leisure and social functions.
- Technological integration: 4/4. Full configuration of Wi-Fi, power outlets, multimedia equipment, and information terminals.
- Environmental support: 4/4. High-quality acoustic, lighting, and thermal control.
- Social support: 3/4. High overall openness, but few dedicated social node designs.

#### 4.1.4 Cross-site summary and instrument reliability

Marked cross-site heterogeneity is evident across the five dimensions. HU Herun Canteen performs best in spatial variability; both HU Herun Canteen and ZT Sunlight Hall achieve full marks in functional versatility; ZN Library Lobby has the most comprehensive technological integration; ZT Sunlight Hall and ZN Library Lobby offer high environmental support; and all three sites perform consistently strong in social support, aligning with the core attributes of flexible public spaces.

Inter-rater reliability (IRR) testing showed an average Cohen's kappa of 0.78 across the 20 indicators and an intraclass correlation coefficient (ICC) of 0.82 for the total score, indicating good inter-observer agreement. Limited by the sample size of study sites in this research, these IRR results serve as exploratory reference values.

It should be noted that as an exploratory assessment tool, this checklist only reports inter-rater reliability in this study. Full construct validity, criterion validity and external validity need to be further verified in future large-sample studies.

Future large-sample studies can further verify the psychometric properties of the instrument.

### 4.2 Questionnaire reliability and sample characteristics

Internal consistency reliability testing yielded Cronbach's α = 0.95 for learning satisfaction, 0.95 for learning engagement, and 0.93 for self-efficacy. All three dimensions had α values above 0.90, indicating excellent internal consistency among items. Cronbach's α reflects the internal homogeneity of items within each dimension, verifying that all items in the same dimension jointly measure the same latent construct. It is a basic reliability indicator for measurement instruments, ensuring that the adapted questionnaire has stable measurement performance in the FPS context.

A total of 332 questionnaires were distributed across the three case study sites, and 296 valid responses were returned, yielding a valid response rate of 89.2%. Inclusion criteria for questionnaire participants were: (1) full-time undergraduate and graduate students enrolled at the university; (2) having used the target site at least twice within the past month; and (3) voluntary participation in the survey. The demographic characteristics of the sample are presented in Table 4.

**Table 4:** Demographic characteristics of the survey respondents (*N* = 296).

Variable	Category	Frequency (*N*)	Percentage (%)
Gender	Male	128	43.24
	Female	168	56.76
Age	18–22 years	240	81.08
	23–25 years	43	14.53
	26 years or above	13	4.39
Education	Undergraduate	249	84.12
	Postgraduate	47	15.88
Discipline	Humanities and social sciences	126	42.57
	Science and engineering	118	39.86
	Arts	52	17.57
Grade	Freshman	90	30.41
	Sophomore	92	31.08
	Junior	71	23.99
	Senior or above	43	14.52
Space	ZT sunlight hall	102	34.46
	ZN LIBRARY	98	33.11
	HU Herun canteen	96	32.43

### 4.3 Descriptive characteristics of informal learning experiences

Overall, students' informal learning experiences in highly flexible public spaces are at a moderately high level, with mean scores above 3.88 across all three core dimensions.

#### 4.3.1 Learning satisfaction of students in FPS

Learning satisfaction is generally at a relatively high level (see Table 5). The highest-scoring items cluster around environmental comfort and facility accessibility, consistent with the descriptive association between spatial environmental support and technological integration and students' learning experiences. The relatively lower-scoring items are ability to focus without distraction and availability of group discussion zones, indicating that there remains room for improvement in acoustic environment management and functional zoning. This finding is also corroborated by interviewees' reports of “noise disruption during meal hours” in the qualitative data.

**Table 5:** Descriptive statistics of students' learning satisfaction in FPS.

Item	Strongly disagree *N* (%)	Disagree *N* (%)	Neutral *N* (%)	Agree *N* (%)	Strongly agree *N* (%)	Mean	Std. dev.
This space provides a comfortable learning environment.	3 (1.01)	9 (3.04)	30 (10.14)	150 (50.68)	104 (35.14)	4.31	0.74
Opening hours meet my learning needs.	4 (1.35)	11 (3.72)	32 (10.81)	152 (51.35)	97 (32.77)	4.28	0.76
Facilities in the space are convenient to use.	5 (1.69)	12 (4.05)	34 (11.49)	149 (50.34)	96 (32.43)	4.24	0.79
I am satisfied with the overall experience.	4 (1.35)	13 (4.39)	36 (12.16)	150 (50.68)	93 (31.42)	4.18	0.81
The space helps me concentrate without disturbance.	7 (2.36)	22 (7.43)	48 (16.22)	140 (47.30)	79 (26.69)	3.92	0.88
I can easily find an area suitable for group discussion.	6 (2.03)	21 (7.09)	50 (16.89)	142 (47.97)	77 (26.02)	3.95	0.87

#### 4.3.2 Learning self-efficacy of students in FPS

Self-efficacy is overall at a moderately high level (see Table 6). Items related to routine learning tasks receive higher scores, indicating that flexible public spaces provide relatively good support for basic learning tasks. Items related to higher-order cognition scores comparatively lower, suggesting that the sites provide insufficient support for complex deliberation and challenging learning activities.

**Table 6:** Descriptive statistics of students' self-efficacy in FPS.

Item	Strongly disagree *N* (%)	Disagree *N (*%)	Neutral *N* (%)	Agree *N* (%)	Strongly agree *N* (%)	Mean	Std. dev.
Studying in this space helps me better understand learning content.	4 (1.35)	12 (4.05)	37 (12.50)	149 (50.34)	94 (31.76)	4.19	0.80
I feel more confident about completing learning tasks.	5 (1.69)	13 (4.39)	40 (13.51)	150 (50.68)	88 (29.73)	4.14	0.82
This space helps me arrange my learning pace autonomously.	5 (1.69)	15 (5.07)	41 (13.85)	148 (50.00)	87 (29.39)	4.11	0.84
Studying in this space makes me more willing to try challenging tasks.	8 (2.70)	23 (7.77)	57 (19.26)	137 (46.28)	71 (23.99)	3.88	0.90
This space facilitates discussions of difficult questions with peers.	7 (2.36)	21 (7.09)	55 (18.58)	138 (46.62)	75 (25.34)	3.91	0.89

This pattern is directly related to the inherent dimensional attributes of FPS, which is further corroborated by interviewee feedback.

#### 4.3.3 Learning engagement of students in FPS

Learning engagement exhibits a clear internal stratification pattern: the behavioral engagement dimension scores highest, followed by affective engagement, with cognitive engagement scoring the lowest (see Table 7). This result indicates that the association between flexible public spaces and students' learning investment is most pronounced at the behavioral level, while there remains considerable room for improvement in fostering deep cognitive processing. This finding aligns with conclusions from similar studies of Chinese universities: public-derived informal spaces have a notable behavioral driving effect on learning, but their support for deep cognitive engagement is limited by the functional boundaries of the space (Wang and Han, 2021). Interviewee remarks that “the open atmosphere easily motivates study, but for deep thinking we go to quiet areas” echo the quantitative findings.

**Table 7:** Descriptive statistics of students' learning engagement in FPS.

Item	Strongly disagree *N* (%)	Disagree *N* (%)	Neutral *N* (%)	Agree *N* (%)	Strongly agree *N* (%)	Mean	Std. dev.
I often use this space for studying.	2 (0.68)	10 (3.38)	32 (10.81)	150 (50.68)	102 (34.46)	4.29	0.72
I spend a long time studying in this space.	4 (1.35)	13 (4.39)	39 (13.18)	148 (50.00)	92 (31.08)	4.17	0.78
I enjoy studying in this space.	4 (1.35)	12 (4.05)	42 (14.19)	150 (50.68)	88 (29.73)	4.23	0.79
Studying in this space increases my interest in learning.	6 (2.03)	18 (6.08)	49 (16.55)	146 (49.32)	77 (26.02)	3.98	0.88
I actively think about how to connect knowledge.	6 (2.03)	20 (6.76)	53 (17.91)	145 (48.99)	72 (24.32)	3.93	0.90
I try different ways to understand learning content.	7 (2.36)	23 (7.77)	56 (18.92)	140 (47.30)	70 (23.65)	3.88	0.91

The behavioural engagement dimension comprises items 1, 2, the affective engagement dimension comprises items 3, 4, and the cognitive engagement dimension comprises items 5, 6.

#### 4.3.4 Correlation analysis of the three learning experience dimensions

To address RQ3, Spearman's rank correlation analysis was conducted to examine the overall associative pattern among the three dimensions. Results reveal significant strong positive correlations between all pairs (*p* < 0.001): the correlation coefficient between learning satisfaction and learning engagement is *r*_*s*_ = 0.742; between learning satisfaction and self-efficacy, *r*_*s*_ = 0.758; and between self-efficacy and learning engagement, *r*_*s*_ = 0.736 (see Table 8 for details).

**Table 8:** Correlation analysis of learning satisfaction, self-efficacy, and learning engagement.

Variable	Learning satisfaction	Learning engagement	Self-efficacy
Learning satisfaction	1.00	0.742^***^	0.758^***^
Learning engagement		1.00	0.736^***^
Self-efficacy			1.00

^***^p < 0.001; Spearman's correlation analysis was used.

These findings indicate that students' informal learning experiences are highly holistic, and the three dimensions form a mutually reinforcing experiential pattern. The perceived chain described by interviewees, “environmental satisfaction → boosted confidence → greater willingness to invest in learning”, provides qualitative corroboration of the quantitative correlation results.

#### 4.3.5 Descriptive comparison of experiences across case study sites

As an exploratory descriptive analysis, cross-site comparison aims to present the trend of experiential differences across different types of FPS, provide preliminary descriptive evidence for subsequent large-sample verification studies, and offer a reference for differentiated optimization of different public spaces. Given the convenience sampling design, no statistical inferences about causal relationships are drawn.

Descriptive comparisons of learning satisfaction, self-efficacy, and learning engagement across the three case sites are presented in Table 9. Numerically, ZT Sunlight Hall achieves slightly higher scores on all three measures than the other two sites, while HU Herun Canteen scores the lowest. As this study employed convenience sampling and the three groups were non-randomly assigned, the observed mean differences are purely descriptive. They cannot be attributed to differences in flexible public space characteristics, and no statistical inferences are drawn.

**Table 9:** Comparison of mean experience scores across case study sites (*M* ±*SD*).

Case site	Learning satisfaction	Self-efficacy	Learning engagement
ZT sunlight hall	4.21 ± 0.75	4.17 ± 0.77	4.20 ± 0.71
HU Herun canteen	4.08 ± 0.83	4.02 ± 0.84	4.07 ± 0.79
ZN library lobby	4.16 ± 0.78	4.10 ± 0.79	4.14 ± 0.73

### 4.4 Qualitative analysis: subjective perception pathways and optimization demand

Thematic analysis of the seven interview transcripts yielded three subjective experiential pathways perceived by students and one core set of inherent tensions, all of which align with the quantitative findings. The three overarching themes correspond respectively to the three core variables of the quantitative study: spatial atmosphere catalysis maps onto learning satisfaction, autonomy enhancement maps onto self-efficacy, and social interaction triggering maps onto learning engagement, achieving methodological triangulation between quantitative results and qualitative findings. Basic information about interview participants is presented in Table 10, and a summary of the thematic analysis is provided in Table 11.

**Table 10:** Basic information about the interviewees.

ID	Grade	Discipline	Case space
P01	Sophomore	Humanities and social sciences	HU Herun canteen
P02	Junior	Science and engineering	ZN library
P03	Sophomore	Science and engineering	ZT sunlight hall
P04	First-year postgraduate	Humanities and social sciences	ZT sunlight hall
P05	Junior	Science and engineering	HU Herun canteen
P06	Junior	Arts	ZN library
P07	Second-year postgraduate	Humanities and social sciences	ZT sunlight hall

P, participant.

**Table 11:** Summary of thematic analysis: student experiences with FPS.

Type	Theme	Subtheme	Corresponding quantitative dimension	Example quote	Frequency
Core perceived pathways	Catalytic effect of spatial atmosphere	Infection by others' learning behaviour	Learning satisfaction	Many people study in the library hall. The “everyone is studying” atmosphere influences me and helps me focus better. (Participant 5, ZN Library)	7
		Environmental comfort and openness	Learning satisfaction	The Sunlight Hall has natural light and is very spacious. Studying here makes me feel relaxed. (Participant 4, ZT Sunlight Hall)	5
	Enhancing effect of autonomy	Flexible choice of study location	Self-efficacy	The advantage of HU Herun Canteen is that I can choose my own spot. I can sit in the corner for quiet study or at the large central table for discussion. (Participant 6, HU Herun Canteen)	6
		Sense of control over the learning process	Self-efficacy	Being able to decide where to study makes me feel more proactive about learning. (Participant 6, HU Herun Canteen)	4
	Triggering effect of social interaction	Peer encounter and spontaneous discussion	Learning engagement	Once I studied in the Sunlight Hall and met a classmate from the same course. We talked about assignments and later often studied here together. (Participant 7, ZT Sunlight Hall)	5
		Opportunities for collaborative learning	Learning engagement	Open seating makes it easier for people to discuss questions. (Participant 3, ZT Sunlight Hall)	3
Potential tensions	Tension between openness and focus	Noise disturbance in open spaces	—	The atmosphere at HU Herun Canteen is nice, but sometimes it is too lively. I would not choose this place if I needed to concentrate on reading academic papers. (Participant 1, HU Herun Canteen)	4
		Need for quiet and private spaces	—	If we need to have long discussions or deep thinking, we usually go to a study room. (Participant 2, ZN Library)	3

“Frequency of mention” refers to the number of coded references corresponding to the theme across all interview transcripts; it does not indicate the magnitude of effect.

Interviews also identified clear optimization demands, including zoned spatial layout, acoustic environment improvement, additional power outlets, and increased discussion booths, providing first-hand evidence for the optimization of campus public spaces.

## 5 Discussion

### 5.1 The quality of students' learning experiences in flexible public spaces

This study finds that students' overall informal learning experiences in the three highly flexible public spaces are at a moderately high level, and learning engagement exhibits a clear stratified pattern characterized by high behavioral engagement and low cognitive engagement. This finding can be explained from the perspective of alignment between spatial attributes and learning needs:

First, functional versatility serves as the core appeal of such spaces, but it also inherently limits support for deep cognitive processing. With diverse original functions, public spaces cannot be designed with the same level of strict acoustic isolation and spatial partitioning as dedicated study rooms. Accordingly, they are better suited to low-focus, surface-level learning scenarios such as routine independent study and social interaction and have inherent limitations in supporting higher-order cognitive tasks that require deep concentration and complex deliberation. This characteristic essentially reflects students' general dual demand for informal spaces: students require both open social interaction settings and private focused study spaces, and the two cannot be simultaneously maximized within a single undifferentiated space (Zhang et al., 2022). This pattern is common across cross-cultural contexts and is not unique to universities in mainland China. This finding aligns with the Hong Kong case study by Chin et al. (2021) and the conclusions of a systematic review covering 14 countries by Salih et al. (2024): public-derived, non-specialized learning venues such as canteens, student service halls, and library atriums are inherently suited to surface-level informal learning such as basic independent study and simple collaboration. Insufficient support for deep cognitive activities is a shared characteristic of such spaces across contexts with different cultures and levels of economic development.

Research on campus pocket social spaces by Alzamil et al. (2023) also indirectly corroborates this pattern: open public spaces with functional versatility excel at supporting social interaction and routine learning tasks, while their capacity to support deep cognitive activities is constrained by spatial boundaries and acoustic conditions. Hunter and Cox (2014) further note that campus public spaces with traditional functional orientations generally face the issue of insufficient higher-order cognitive support during their transformation into learning spaces.

Regarding instrument quality, the locally adapted questionnaire demonstrates excellent internal consistency reliability (α > 0.90), and the spatial evaluation checklist shows good inter-rater reliability (Kappa = 0.78, ICC = 0.82). However, both are exploratory instruments that have not undergone full construct validation, and further optimization is required in future research.

### 5.2 Mutually reinforcing associations among the three dimensions of learning experience

Spearman correlation analysis reveals strong positive pairwise correlations among learning satisfaction, self-efficacy, and learning engagement, which supports and extends prior research findings within the context of flexible public spaces (Carini et al., 2006; Shea and Bidjerano, 2010). The three dimensions form a positive cycle in students' subjective perceptions: a spatial environment that meets needs elevates satisfaction, which in turn strengthens self-efficacy and ultimately promotes learning investment; the positive feelings generated by learning investment in turn reinforce both satisfaction and self-efficacy.

Qualitative analysis identifies the enabling conditions for this cycle: spatial atmosphere provides the foundation for satisfaction, flexible choice enhances self-efficacy, and open layout drives social engagement. This finding suggests that future research should adopt a holistic perspective, exploring how physical spaces may shape learning behaviors by influencing affective evaluations and ability beliefs. It should be noted that as this study employs a cross-sectional design, this cycle represents an experiential pattern self-reported by students and does not constitute verified causal relationships.

### 5.3 Students perceived experiential pathways and theoretical dialogue

Three experiential pathways perceived by students were inducted from the interviews, which can be brought into dialogue with classic theories and cutting-edge research. The perceived catalytic effect of spatial atmosphere echoes the concept of “situational contagion” in environmental psychology (Barsade, 2002), whereby individual behavior is influenced by the overall atmosphere of the environment, consistent with findings that students are influenced by the learning behavior of others, leading to increased concentration; the perceived enhancing effect of autonomy aligns with the view in self-determination theory that “the fulfillment of autonomy needs can enhance individual motivation and a sense of efficacy” (Ryan and Deci, 2000), explaining why flexible spatial choices can enhance students' sense of control over their learning; whilst the perceived triggering effect of social interaction embodies the social constructivist view of learning (Vygotsky, 1978), namely that learning is a process of knowledge construction achieved through social interaction. This finding is consistent with the functional logic of pocket social learning spaces proposed by Alzamil et al. (2023): open, flexible small-scale social nodes can effectively trigger spontaneous learning interactions among students, serving as an important complement to formal learning. This conclusion also further validates the field consensus, established in Salih et al.'s (2024) typology, that the social attribute of public spaces is a core element of learning support.

Meanwhile, this study identifies a core tension in the design of flexible public spaces: the contradiction between openness and concentration. Flexibility is not equivalent to undifferentiated open design. Truly effective FPS should incorporate adjustability and selectivity, allowing users to autonomously switch between social mode and quiet mode according to their current task. This aligns with the perspective of “space as a socio-spatial assemblage” proposed by Dovey and Fisher (2014).

### 5.4 Optimization directions for flexible public spaces informed by student feedback

Integrating into the higher education learning space restructuring framework proposed by Elkington and Dickinson (2025) and based on student feedback from qualitative interviews, the optimization of university FPS can be systematically advanced across three dimensions: spatial design, technical support, and management, thereby addressing RQ4. Li et al. (2025) also corroborate this bottom-up optimization logic based on evidence from Japanese universities, noting that repurposing existing spaces and extending opening hours can effectively improve space utilization without new construction.

#### 5.4.1 Spatial design

Student feedback demand: interviews revealed that students clearly reported noise disturbance in open areas and a strong demand for functional zoning, expecting a clear separation between quiet study areas and group discussion areas.

Optimization direction: a zoned layout is established, dividing the open space into “active zones” and “quiet zones”, with acoustic treatments and flexible partitions employed to minimize mutual disturbance; Movable furniture and reconfigurable layouts are retained to preserve spatial adaptability, allowing students to arrange the space according to their own needs; while dedicated social hub nodes are set up to encourage chance encounters and interaction, ensuring these do not encroach upon areas dedicated to focused study.

Theoretical support: this design responds to the dual preference for privacy and interaction in informal learning spaces proposed by Zhang et al. (2022) and aligns with the perspective of “space as a socio-spatial assemblage” proposed by Dovey and Fisher (2014), which emphasizes both spatial convertibility and functional fluidity of space.

#### 5.4.2 Technical support

Student feedback demand: students generally reported insufficient power sockets in partial areas and a lack of multimedia equipment in social interaction nodes, which restricted the completion of collaborative learning tasks.

Optimization direction: comprehensive stable Wi-Fi coverage is ensured, power sockets are evenly distributed across functional zones, and multimedia display devices are added at social hubs to support diverse collaborative learning methods.

Theoretical support: this configuration aligns with the technical configuration standards of smart learning spaces proposed by El Kiki et al. (2015), which emphasizes that adequate infrastructure supports active learning and collaboration in learning spaces.

#### 5.4.3 Management mechanisms

Student feedback demand: students mentioned space use conflicts during peak hours and expected a smooth channel to feedback spatial usage problems and optimization suggestions.

Optimization direction: regular student participation and feedback channels are established, and student experience data are incorporated into spatial renewal decision-making, forming a closed loop of “assessment–feedback–optimization” to enable continuous iteration and improvement of FPS.

Theoretical support: this bottom-up iterative mechanism is consistent with the higher education learning space restructuring framework proposed by Elkington and Dickinson (2025), which emphasizes the integration of user experience into the full lifecycle of space design and operation.

## 6 Conclusion

Taking four universities in Zhejiang Province as case sites, this study adopts explanatory sequential mixed methods designed to systematically investigate the characteristics of students' informal learning experiences in highly flexible public spaces and their underlying perceived pathways. The main conclusions and contributions are drawn as follows.

### 6.1 Core findings

Firstly, the characteristics of university FPS can be summarized in terms of five core dimensions: spatial variability, functional versatility, technological integration, environmental support and social support. The three representative spaces identified using this framework (ZT Sunlight Hall, HU Herun Canteen and ZN Library Lobby) all demonstrate a high degree of flexibility (80%−90%) and can support a wide range of learning activities.

Second, students' overall IL experiences in FPS are at a moderately high level, but the cognitive engagement dimension is comparatively low. This indicates that such venues still provide inadequate support for higher-order deep learning.

Third, strong positive pairwise correlations exist among learning satisfaction, self-efficacy, and learning engagement, which form a mutually reinforcing positive experiential cycle in students” subjective perceptions.

Fourth, students subjectively perceive three experiential pathways: atmosphere catalysis, autonomy enhancement, and social triggering. Meanwhile, an inherent tension exists between spatial openness and learning concentration, suggesting that the design of flexible public spaces needs to strike a balance between multifunctionality and targeted functional demands.

### 6.2 Theoretical and practical contributions

At the instrument level, this study develops a five-dimensional, 20-item FPS evaluation checklist with preliminary inter-rater reliability verification, providing an operational quantitative tool for campus public space assessment and addressing the gap of operational flexibility measurement tools for Chinese university public spaces. At the empirical level, grounded in mainland Chinese university contexts, the study provides systematic mixed-methods evidence for multi-dimensional informal learning experiences in highly flexible public spaces, expanding the empirical base of East Asian research in this field beyond existing Hong Kong and Southeast Asian case studies.

At the theoretical level, the study identifies the inherent tension between openness and focus on FPS, induces three subjective experiential pathways, and establishes dialogue with environmental psychology, self-determination theory and social constructivist learning theory, offering a perceptual interpretation framework for students' experiences in public spaces.

In practice, the findings provide a bottom-up evidence base for the functional optimization of campus public spaces and the upgrading of learning support systems in higher education. The three-dimensional optimization framework of spatial design, technical support and management mechanisms offers actionable references for university spatial governance.

### 6.3 Limitations and future directions

This study has several limitations. First, it is a cross-sectional investigation that only includes highly flexible public spaces as cases, without medium- and low-flexibility control groups. Accordingly, it does not examine the association between the level of spatial flexibility and learning outcomes and only describes the experiential characteristics within highly flexible FPS. It can only reveal statistical associations among experience variables rather than causal relationships.

Second, both the evaluation checklist and the adapted questionnaire are exploratory instruments that have not undergone full construct validation. Third, the sample is limited to four universities in Zhejiang Province using convenience sampling, resulting in limited external generalizability.

Future research can advance across multiple dimensions: further refine and validate measurement tools with large-sample data; adopt longitudinal tracking designs and add high/low-flexibility control groups to more rigorously examine the associations between spatial characteristics and learning experiences; and explore differentiated demands for FPS among different student groups, to gradually build a complete ecosystem of campus learning spaces.

## Data Availability

The datasets presented in this article are not readily available because the dataset contains sensitive qualitative interview data and quantitative questionnaire data involving human participants. According to the informed consent and institutional guidelines, the raw data cannot be shared publicly or upon request. Only aggregated, anonymized findings are provided in the article. Requests to access the datasets should be directed to Juncheng Yang, 193194770@qq.com.
