# Supplementary material for: Characteristics and perceived pathways of students' informal learning experiences in highly flexible public spaces of higher education: a mixed-method study based on four universities in Zhejiang Province
Source: Front Psychol. 2026 Oct 20;17:1882283. doi: 10.3389/fpsyg.2026.1882283 (PMC13727644; doi:10.3389/fpsyg.2026.1882283)
Supplement: Supplementary file 1 [file Data_Sheet_1.pdf]

Supplementary Material

1 Appendices

Appendix A: Questionnaire Sample Items

A1: Student demographic information

(1) Please state your gender.

(2) Please state your age group.

(3) Please state your level of education.

(4) Please state your field of study.

(5) Please state the year you are in.

A2: Student satisfaction with studying in flexible public spaces

Please rate your level of agreement with the following statements regarding your satisfaction with studying in this space. (1 = Strongly disagree; 2 = Disagree; 3 = Neutral; 4 = Agree; 5 = Strongly agree)

(1) This space provides a comfortable learning environment.

(2) The opening hours of the space meet my study needs.

(3) The facilities within the space (tables, chairs, power sockets, etc.) are convenient to use.

(4) This space helps me to concentrate on my studies without being disturbed.

(5) I can easily find areas suitable for group discussions.

(6) Overall, I am satisfied with my study experience in this space.

A3: Students' self-efficacy in learning within flexible public spaces

Please rate your level of agreement with the following statements regarding your self-efficacy in learning within this space. (1 = Strongly disagree; 2 = Disagree; 3 = Neutral; 4 = Agree; 5 = Strongly agree)

(1) When learning in this space, I am able to understand the learning content better.

(2) I feel more confident about completing my learning tasks.

(3) This space helps me to organise my own learning pace.

(4) When studying in this space, I am more willing to attempt challenging learning tasks.

(5) This space helps me to discuss difficult questions with my classmates.

(6) Overall, I am confident in my ability to complete learning tasks in this space.

A4: Student engagement in flexible communal spaces

Please rate your level of agreement with the following statements regarding your engagement with learning in this space. (1 = Strongly disagree; 2 = Disagree; 3 = Neutral; 4 = Agree; 5 = Strongly agree)

(1) I frequently use this space for studying.

(2) I spend a considerable amount of time studying in this space.

(3) I enjoy studying in this space.

(4) Studying in this space makes me more interested in learning.

(5) When studying in this space, I actively think about how to connect different pieces of knowledge.

(6) I try to understand the learning material in different ways.

A5: Interview Invitation

We would like to invite you to take part in an interview to discuss your learning experience in this space in more detail.

Please indicate whether you would be willing to participate.

☐ Yes, I am interested. ☐ No, I am not interested.

Thank you for agreeing to take part in the interview.

Please provide your name and email address below.

Appendix B: Interview Guide

(1) How would you describe this space as a place for learning?

(2) Please tell me about your experience of using this space for learning activities (satisfaction, engagement, confidence). Could you elaborate by referring to a specific activity or occasion?

(3) What factors influence your choice of this space for learning?

(4) What feelings or thoughts do you have when studying in this space?

(5) Which specific aspects of this space support your learning? Which aspects might hinder your learning?

(6) How does this space compare to other learning spaces on campus?

(7) Based on your experience, what are the benefits of flexible public spaces such as this for learning? Could you elaborate by referring to a specific activity?

(8) Do you have any suggestions for improving this space to better support student learning?
